# Construction of Microsphere Culture System for Human Mesenchymal Stem Cell Aggregates

**DOI:** 10.3390/ijms26136435

**Published:** 2025-07-04

**Authors:** Chenlong Lv, Shangkun Li, Min Sang, Tingting Cui, Jinghui Xie

**Affiliations:** 1 State Key Laboratory of Pathogenesis, Prevention and Treatment of High Incidence Diseases in Central Asia, School of Basic Medical Sciences, Xinjiang Medical University, Urumqi 830000, China; chenlonglv@126.com (C.L.); lishangkun2025@163.com (S.L.); comqqsangmin@163.com (M.S.); 2 Xinjiang Key Laboratory of Molecular Biology for Endemic Diseases, School of Basic Medical Sciences, Xinjiang Medical University, Urumqi 830000, China

**Keywords:** mesenchymal stem cells, alginate hydrogel, cell aggregate, electrostatic spraying technology

## Abstract

Stem cells cultured in cell aggregates exhibit higher cell survival rates and enhanced anti-inflammatory and angiogenic effects compared to single cells, constructing a stable and economical cell aggregate culture system that can accurately adjust the mass transfer distance of nutrients, which contributes to improving the therapeutic effects of stem cell aggregates. In this study, an alginate hydrogel microsphere culture system (Alg-HM) was prepared using electrostatic spraying technology and refined by optimizing the electrostatic spraying technology parameters, such as the sodium alginate concentration, voltage, electrospray injection speed, and nozzle inner diameter. Furthermore, by setting the Tip-dropped culture system (Tip-D culture system, created by dropping the resuspended hMSC aggregate–hydrogel solution with a tip to form the hydrogel microsphere) and Matrigel culture system (created by dropping the resuspended hMSC aggregates–Matrigel solution with a tip to form the Matrigel culture system) as the control group and Alg-HM as the experimental group, the culture effect of hMSC aggregates in the optimized Alg-HM culture system was tested; CCK-8 detection and Ki-67 immunofluorescence staining showed that the Alg-HM culture system significantly enhanced the cell proliferation activity of hMSC aggregates after 7 and 14 days of culture. The Calcein-AM/PI cell staining results showed that the Alg-HM culture system can significantly reduce the central necrosis of hMSC aggregates. The RNA sequencing results showed that the Alg-HM culture system can significantly activate the signaling pathways related to cell proliferation in hMSCs. This culture system is helpful for the culture of cell aggregates in vitro and efficient transplantation in vivo.

## 1. Introduction

The specific differentiation and cell factor secretion abilities make human mesenchymal stem cells (hMSCs) the most promising stem cell candidate for cell therapy, which has been widely used in the fields of regenerative medicine, autoimmune diseases, and tissue engineering [1,2]. However, single-cell hMSCs transplantation has limitations, such as the low cell survival rate after transplantation [3,4,5]. A large number of studies have shown that hMSCs cultured in cell aggregates exhibit higher cell survival rates and enhanced anti-inflammatory and angiogenic effects, which help enhance their therapeutic effect in vivo [6,7,8]. Maintaining the viability of cell aggregates during cell culture in vivo remains a key step in cell aggregate research. Currently, in addition to hanging drop culture technology, microgravity rotating cell culture systems (RCCSs), microfluidic chip culture technology, and hydrogel-based culture technology have all been used for cell aggregate culture [9,10,11].

Compared to other culture technologies that require special equipment or complicated operation steps, scaffold culture is relatively simple and can simulate a realistic extracellular matrix (ECM) environment for cell aggregates. Thus, Matrigel, hyaluronic acid hydrogel, and alginate hydrogel have been widely used in three-dimensional cell aggregate culture [12,13]. The mass transfer of nutrients is a key factor affecting the cell viability of the aggregate in a hydrogel culture. As the mass transfer distance within the hydrogel increases, nutrient diffusion to the center becomes exponentially less efficient, which leads to cell necrosis due to hypoxia and nutrient deprivation [14,15]. Studies have shown that the oxygen concentration is relatively stable when the radius of the hydrogel is less than 200 μm; a greater mass transfer distance means that the concentration of nutrients is higher in the outer layer and lower in the center of the hydrogel, eventually leading to central cell necrosis [16,17]. However, conventional hydrogel culture simply mixes the cell aggregates with hydrogels and cannot accurately control the transport distance of nutrients. For instance, in a Tip-dropped culture system (Tip-D culture system, created by dropping the resuspended hMSC aggregate–hydrogel solution with a tip to form the hydrogel microsphere), the smallest diameter of the hydrogel microspheres dripped out is larger than a few millimeters, and the particle size of the microspheres cannot be precisely controlled. Precisely controlling the nutrient transport distance significantly improves the cell viability of cell aggregates in hydrogel culture.

Unlike traditional hydrogel-based culture, studies have shown that hydrogel microsphere culture systems can precisely control the distance of material transmission under culture conditions and show higher nutrient permeability [18,19]. Several studies have demonstrated the advantage of alginate hydrogel microspheres in culturing dispersed cells, showing improved cell viability, less heterogeneity, and enhanced drug metabolic activity [20,21,22]. This microsphere culture system helps shorten, adjust, and control the mass transfer distance within hydrogels, ultimately improving the cell survival rate; however, until now, there is no research on the culture of cell aggregates via a microsphere culture system. Therefore, in this study, a cell aggregate microsphere culture system based on alginate hydrogel material was constructed (alginate hydrogel microsphere, Alg-HM), and the method for preparing microspheres suitable for cell aggregate culture was optimized.

## 2. Results

### 2.1. The Construction of the Alg-HM Culture System for the Culture of hMSCs

**Figure 1:**
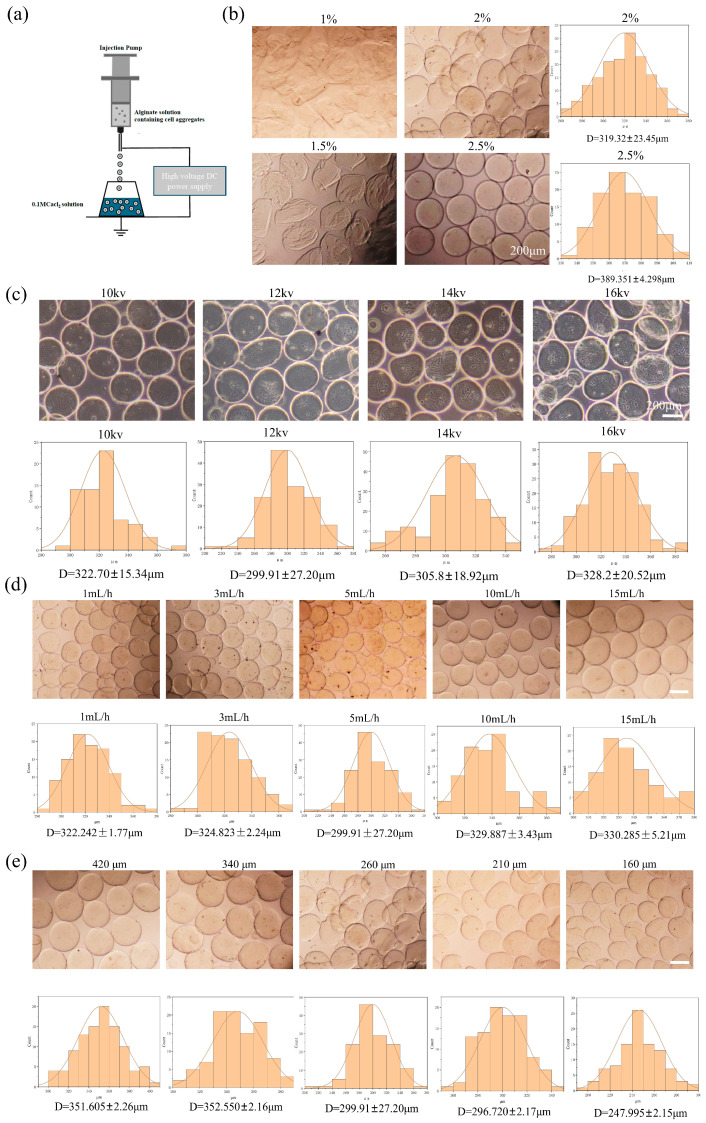
Effects of different parameters on particle size of Alg-HM. (**a**) Model of Alg-HM preparation process, (**b**) Alg-HM prepared by different concentrations of alginic acid solution and its particle size statistics, (**c**) Alg-HM prepared at different voltages and its particle size statistics, (**d**) Alg-HM prepared at different injection rates and its particle size statistics, (**e**) Alg-HM prepared at different nozzle inner diameters and its particle size statistics (scale bar: 200 µm).

In this study, an electrostatic spraying device was constructed using a high-voltage direct current (DC) power supply and a microinjection pump. Alg-HM was prepared by using this device to electrostatically spray a sodium alginate solution (Figure 1a). To construct an Alg-HM culture system that could effectively increase the survival rate of cell aggregates, we optimized the parameters affecting the particle size of Alg-HM, such as the sodium alginate concentration, voltage, injection speed, and nozzle inner diameter. First, the concentration of sodium alginate was optimized, as shown in Figure 1b. We found that low-concentration sodium alginate could not form complete regular microspheres; however, when the concentration of the sodium alginate solution was 2%, Alg-HM demonstrated good spheroidization and a small particle size. With the increase in solution concentration, the particle size of microspheres increased significantly (Figure 1b), which was not beneficial for the transport of nutrients in culture, so a 2% solution was selected for the subsequent preparation of Alg-HM. The influence of voltage on the particle size of Alg-HM was also detected, as shown in Figure 1c; it was found that the voltage has a dual effect on the particle size of microspheres in the range of 10–16 kV. The particle size of microspheres is significantly larger at lower and higher voltages, and the particle size of the prepared Alg-HM microsphere was the lowest at 12 kV (299.91 ± 27.2 μm, Figure 1c). In addition, the solution injection speed was also optimized, as shown in Figure 1d; the particle size of the prepared Alg-HM gradually decreased with the increase in injection speed in the range of 1–5 mL/h. However, when the injection rate was over 5 mL/h, the particle size of the prepared Alg-HM gradually increased in the range of 5–15 mL/h (Figure 1d); thus, 5 mL/h was selected for the subsequent preparation of Alg-HM.

The nozzle inner diameter was also optimized; the nozzles with inner diameters of 420 μm, 340 μm, 260 μm, 210 μm, and 160 μm were selected. Among them, we found that when the inner diameter of the nozzle was less than 160 μm, the cell aggregates could not be ejected smoothly. As shown in Figure 1e, when the nozzle inner diameter was 160 μm, the particle size of the Alg-HM was significantly smaller. According to these results, a 2% sodium alginate solution, a voltage of 12 kV, a 5 mL/h injection speed, and a 160 μm nozzle inner diameter were chosen for the preparation of Alg-HM.

### 2.2. The Alg-HM Culture System Significantly Reduces the Central Necrosis and Increases the Proliferation Activity of the hMSC Aggregate

**Figure 2:**
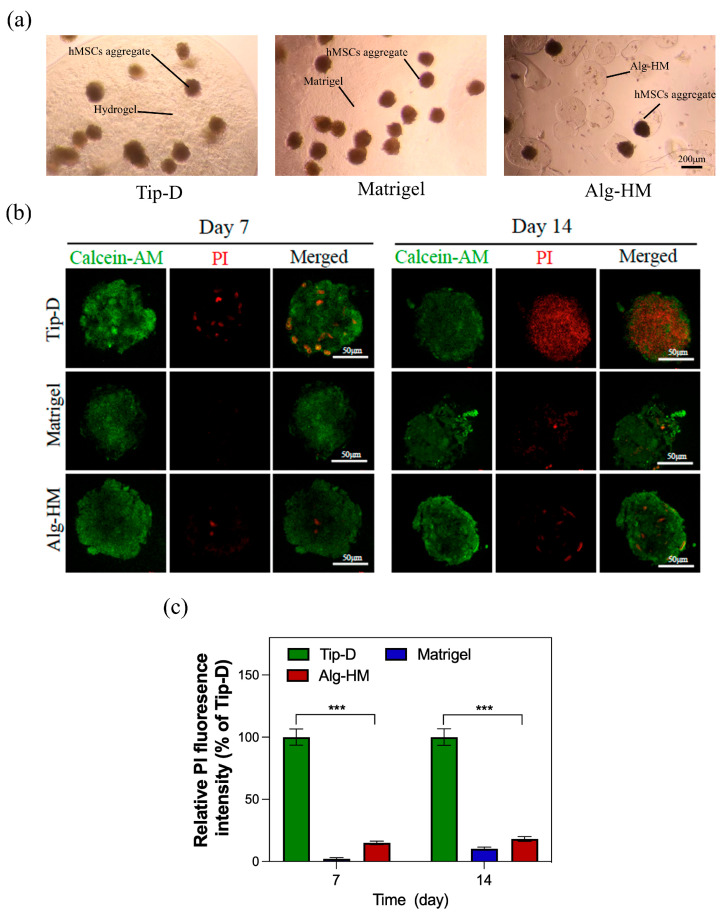
The Alg-HM culture system significantly reduces the degree of central cell necrosis of hMSC aggregates. (**a**) Construction of different hMSC aggregate culture systems (scale bar: 200 µm). (**b**) hMSC aggregates in different culture systems were stained with Calcein-AM (green for live cells) and PI (red for dead cells) at day 7 and day 14 (scale bar: 50 µm). (**c**) Quantification of the fluorescence intensity of PI in each group (n = 5; *** *p* < 0.001 relative to Tip-D).

Then, the optimized Alg-HM culture system was used for the cultivation of the hMSC aggregate (Figure 2a), and Calcein-AM/PI cell staining was used to detect the central necrosis of the cell aggregate. As shown in Figure 2b,c, the hMSC aggregate cultured in the Tip-D culture system showed much more cell death at day 7 and day 14. However, the number of dead cells in the hMSC aggregate in the Alg-HM culture system was significantly lower than in the Tip-D culture system, similar to the Matrigel group.

**Figure 3:**
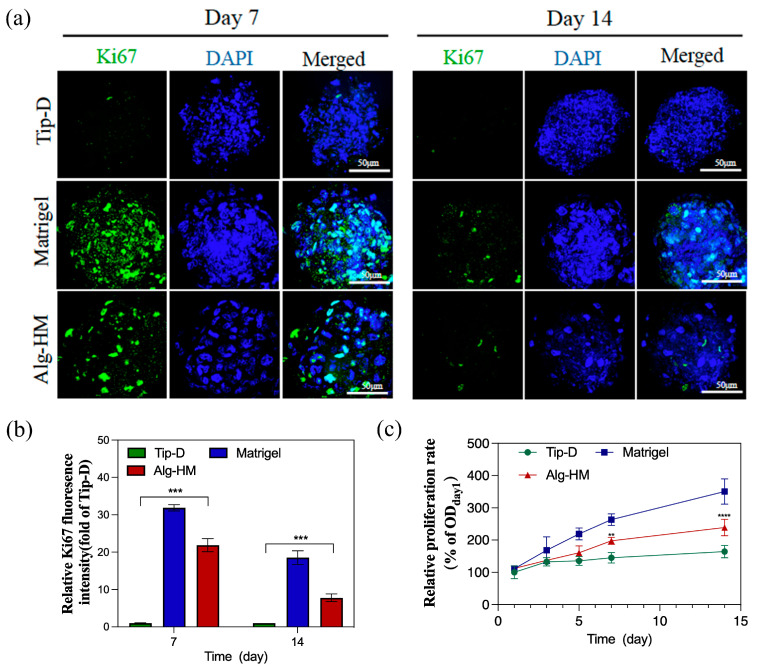
The Alg-HM culture system promotes the proliferation of the hMSC aggregates. (**a**) hMSC aggregates in different culture systems were stained with Ki-67 (green for proliferative cells) and DAPI (blue) at day 7 and day 14 (scale bar: 50 µm). (**b**) Quantification of the fluorescence intensity of Ki67 in each group. (**c**) Proliferation of hMSC aggregates in different groups detected via CCK-8 at days 1, 3, 5, 7, and 14 (n = 5; ** *p* < 0.01, *** *p* < 0.001 and **** *p* < 0.0001 relative to Tip-D).

In addition, Ki-67 immunofluorescence staining was used to detect the cell proliferation of hMSC aggregates cultured in different culture systems, as shown in Figure 3a,b; the number of Ki-67^+^ cells in the hMSC aggregate of the Alg-HM culture system was significantly higher than in the Tip-D culture system. The cell viability in the hMSC aggregate was detected via tCCK-8 detection, as shown in Figure 3c; it was found that the cell viability of hMSC aggregates cultured in Matrigel was significantly higher than that of Tip-D and Alg-HM. Moreover, on the 5th, 7th, and 14th days of culture, the cell viability of the hMSCs cultured in Alg-HM was significantly higher than that of the hMSCs cultured in Tip-D (Figure 3c).

### 2.3. The RNA Sequencing Results Showed That the Alg-HM Culture System Can Significantly Activate the Signaling Pathways Related to Cell Proliferation in hMSCs

**Figure 4:**
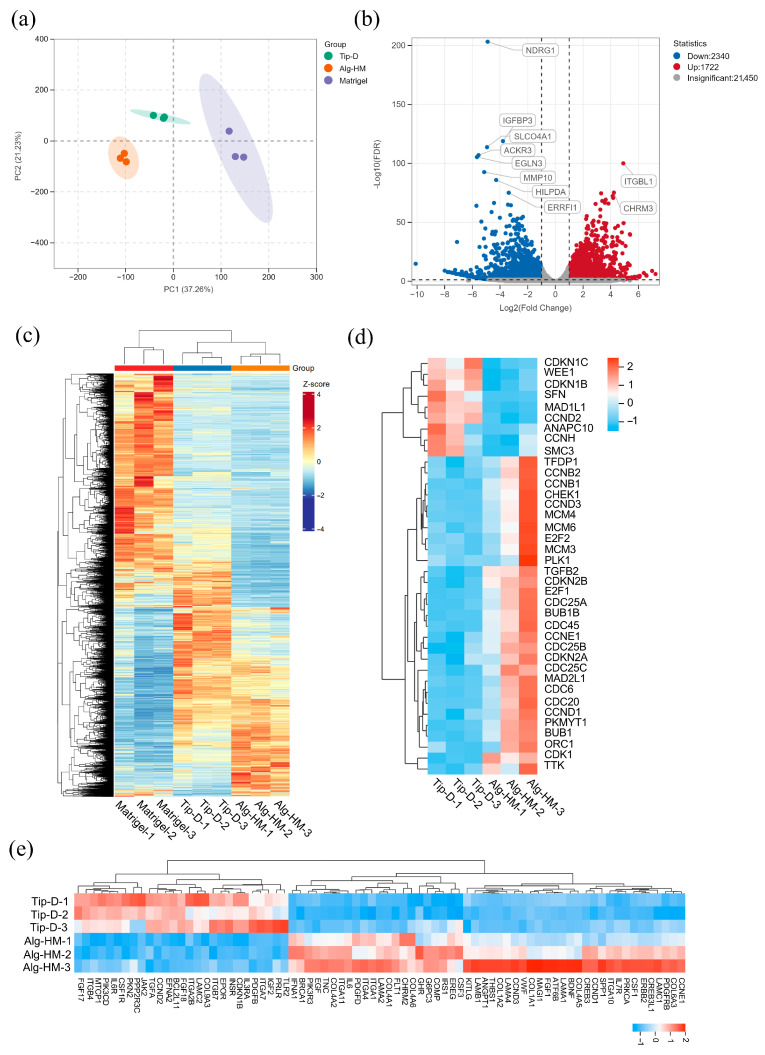
The RNA sequencing results revealed that the Alg-HM culture system can significantly alter the transcriptome of hMSCs in 7-day culture. (**a**) PCA of the transcriptomes in different groups. (**b**) A volcano map showing differentially expressed genes (DEGs) of hMSCs cultured in the Alg-HM culture system compared to the Tip-D culture system. RNA-seq analysis of the transcriptomes of hMSCs cultured for 7 days, including 2340 up-regulated and 1722 down-regulated genes. (**c**) Hierarchical clustering of DEGs of hMSCs cultured in different culture systems for 7 days. (**d**) Hierarchical clustering of DEGs of hMSCs cultured in different culture systems for 7 days in the cell cycle. (**e**) Hierarchical clustering of DEGs of hMSCs cultured in different culture systems for 7 days in the PI3K-AKT signaling pathway.

Then, RNA-sequencing (RNA-seq) analysis was applied to evaluate the influence of the Alg-HM culture system in regulating the gene expression of hMSCs. As shown in Figure 4a, the transcriptomes of hMSCs in different culture environments were clustered in different regions after principal component analysis (PCA), showing good reproducibility. There were a total of 4062 differentially expressed genes (DEGs) in hMSCs cultured in the Alg-HM culture system compared to the Tip-D culture system, including 2340 up-regulated and 1722 down-regulated genes (Figure 4b). The RNA sequencing results showed that the Alg-HM culture system significantly altered the transcriptome of hMSCs compared to the Tip-D culture system and the Matrigel culture system (Figure 4c). Especially in the cell cycle and its related signaling pathway PI3K-ATK, there are significant differences in gene expression (Figure 4d,e).

**Figure 5:**
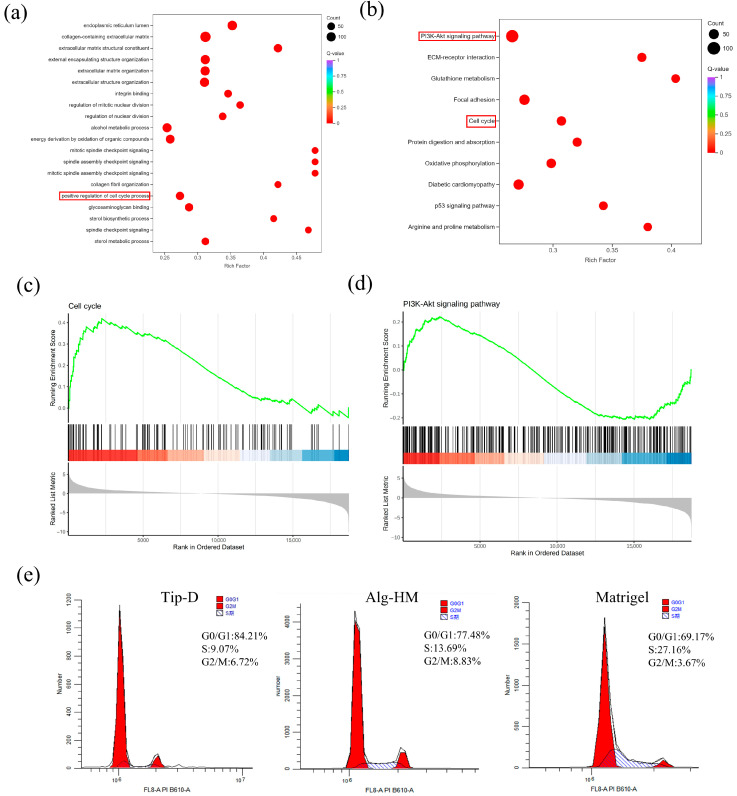
The RNA sequencing results revealed that the Alg-HM culture system can significantly activate the cell signaling pathways related to the cell cycle process in hMSCs in 7-day culture. (**a**) The GO enrichment analysis of the DEGs of Alg-HM vs. Tip-D at 7 days. Red frame: Positive regulation of the cell cycle process. (**b**) The KEGG enrichment analysis of the DEGs of Alg-HM vs. Tip-D at 7 days. Red frame: PI3K-Akt signaling pathway, cell cycle. (**c**) The gene set enrichment analysis (GSEA) of the genes related to the cell cycle of Alg-HM vs. Tip-D at 7 days. (**d**) The gene set enrichment analysis (GSEA) of the genes related to the PI3K-Akt signaling pathway of Alg-HM vs. Tip-D at 7 days. (**e**) The percentage of cells in different phases of the cell cycle assessed via flow cytometry.

Further analysis of cell transcriptomes showed that the Alg-HM culture system can significantly activate the cell signaling pathways related to cell proliferation in hMSCs in 7-day culture (Figure 5). As shown in Figure 5a, GO enrichment analysis of the DEGs at day 7 shows that the Alg-HM culture system has a regulatory effect on the cell cycle process in hMSCs compared to the Tip-D culture system. Gene set enrichment analysis (GSEA) of the genes related to the cell cycle at day 7 also shows that the Alg-HM culture system positively regulates the cell cycle process in hMSCs compared to the Tip-D culture system (Figure 5c and Figure S2). In addition, KEGG enrichment analysis of the DEGs of Alg-HM vs. Tip-D at day 7 shows that the Alg-HM culture system has a regulatory effect on the PI3K-Akt signaling pathway in hMSCs compared to the Tip-D culture system (Figure 5b). Gene set enrichment analysis (GSEA) of the genes related to the PI3K-Akt signaling pathway of Alg-HM vs. Tip-D at day 7 showed that the Alg-HM culture system significantly up-regulated the PI3K-Akt signaling pathway in hMSCs (Figure 5d). The detection of cell cycle distribution was performed via flow cytometry (Figure 5e). The results showed that Alg-HM can reduce the proportion of cells in the G0/G1 phase and increase the proportion of cells in S and G2/M phases, promoting cell cycle progression compared with Tip-D.

## 3. Discussion

More and more studies have shown that stem cells cultured in cell aggregates exhibit higher cell survival rates and enhanced anti-inflammatory and angiogenic effects, which greatly aid tissue repair after transplantation in vivo. However, the maintenance of cell viability in the cell aggregate during in vitro culture remains a key step in the study of cell aggregates. Constructing a stable and economical cell aggregate culture system that can accurately adjust the mass transfer distance of nutrients helps improve the cell proliferation activity of hMSC aggregates, which can facilitate the culture of stem cell aggregates and efficient transplantation in vivo [23,24]. Matrigel is derived from the extracellular matrix and contains a variety of cell adhesion sites and cytokines, which can better promote cell proliferation. In this study, we found that the proliferation rate of the hMSC aggregates cultured in Matrigel was much higher than that of the Tip-D culture system (as shown in Figure 1 and Figure 3, the mass transfer distance of nutrients is close to 1 mm); this result is consistent with previous research results [25]. Although Matrigel contains a variety of growth factors and ECM components, its composition and content remain unclear. At the same time, different batches of Matrigel will have different effects on cells when used to culture stem cells for drug screening or clinical applications [26,27]. Therefore, to overcome these shortcomings, there have been many studies on cultivating cell aggregates with a single-component hydrogel, such as alginate [28].

In this study, a microsphere preparation device was constructed by combining a high-voltage DC power supply and a microinjection pump (Figure 1a), enabling quick and accurate preparation of Alg-HM with uniform and controllable size. Considering that hMSC aggregates with excessive particle size may lead to core hypoxia and necrosis [29], the particle size of the hydrogel was maintained between 200 and 380 μm by adjusting the concentration of the alginate solution, inner diameter of the nozzle, injection speed, and voltage. When the concentration of the alginate solution is 2%, the voltage is 12 kV, the injection speed is 5 mL/h, the inner diameter of the nozzle is 160 μm, and the microspheres can be stably prepared with the smallest particle size. As expected, we found that, when the hMSC aggregates were cultured in this alginate microsphere culture system, the cell viability and proliferation activity of hMSCs were significantly improved, and the cell necrosis was significantly reduced compared to the hMSC aggregates cultured in the Tip-D culture system (CCK-8 results and Calcein-AM/PI cell staining results). At the same time, GO analysis, KEGG analysis, and GSEA data of transcriptomics also show that the Alg-HM culture system can significantly up-regulate the signal pathways related to cell proliferation (PI3K-Akt signaling pathway) and the cell cycle in hMSCs. Compared to the Tip-D culture system, the mass transfer distance of nutrients in the Alg-HM culture system is reduced from 1 mm to less than 120 μm (Figure 1e). These results indicate that, when using the same culture material (alginate hydrogel), reducing the particle size in the microsphere culture system can significantly improve the activity of hMSC aggregates.

In this study, we also found that the cell activity and proliferation activity of the hMSC aggregates in the Alg-HM culture system were still lower than those in Matrigel. This may be because, compared to Matrigel derived from the mouse basement membrane matrix, the alginate hydrogel used in this study contains very few cell adhesion sites and cytokines [30,31]. At the same time, we found that the center of the hMSC aggregates in Matrigel also showed cell necrosis. These results show that both the reduction in the transport distance of substances and the existence of cell adhesion sites and cytokines in the culture environment play an indispensable role in maintaining cell activity and cell proliferation in the hydrogel culture system. This suggests that properly adding cytokines or modifying the alginate in the gel can improve cell activity and adhesion, thus significantly improving the viability and proliferation of hMSC aggregates in the Alg-HM culture system.

## 4. Materials and Methods

### 4.1. Preparation of the Alg-HM Culture System

First, prepare alginate solutions with different concentrations (the solvent is 0.45% NaCl, and the solute is alginate powder) at 1%, 1.5%, 2%, and 2.5% (*w*/*v*) for sterilization. Then, 0.1 M CaCl_2_ (0.45% NaCl as the solvent and anhydrous CaCl_2_ particles as the solute) was filtered through a sterilization filter. A microsphere preparation device was fabricated by combining a high-voltage DC power supply and a microinjection pump. A certain amount of alginate solution was loaded into a 10 mL syringe, and the inner diameters of the nozzles were 420 μm, 340 μm, 260 μm, 210 μm, and 160 μm. A copper ring with a similar diameter was placed at the bottom of the beaker containing the CaCl_2_ solution. The injection speed of the microinjection pump was set at 1 mL/h, 3 mL/h, 5 mL/h, 10 mL/h, and 15 mL/h. The voltages of the high-voltage DC were set to 10 kV, 12 kV, 14 kV, and 16 kV. The combination of these four parameters was adjusted based on the results. Under a certain combination of electrospray parameters, the alginate solution was injected using a syringe at a uniform speed, quickly dropped into the CaCl_2_ solution through a flat nozzle under the action of a high-voltage electrostatic field, and promptly solidified in the CaCl_2_ solution to form Alg-HM. The microspheres were then moved into a common 24-well plate, 200 Alg-HM particles obtained from each group of parameters were randomly measured by ImageJ-1.54p, and the particle size distribution map was calculated.

### 4.2. Cultivation of hMSC and Preparation of hMSC Aggregates

Human umbilical cord mesenchymal stem cells (National Stem Cell Translational Resource Center) were used in this study and were cultured in DMEM/F12 medium (HyClone, Shanghai, China) containing 10% fetal bovine serum (Life-iLab, Shanghai, China) in a cell incubator containing 5% CO_2_ at 37 °C. The culture medium was changed every two days, and when the cell density was above 80%, the cells were passaged with a 0.25% trypsin-EDTA solution (Vivacell, Shanghai, China). The P3 hMSCs were inoculated into an AggreWell^TM^ 24-well plate (StemCell Technologies, Vancouver, BC, Canada) with a layer of anti-adhesive solution (StemCell Technologies, Vancouver, BC, Canada) at a cell density of 3 × 10^5^ cells per well and cultured for 24 h to form cell aggregates.

### 4.3. Construction of hMSC Aggregate Culture System

Three culture systems were constructed for hMSC aggregates in this study. Preparation of Tip-dropped culture system (Tip-D culture system) (Figure S1): hMSC aggregates were resuspended in the alginate solution to form an hMSC aggregate suspension, dropped with a tip into a 0.1 M CaCl_2_ solution, cross-linked for 3 min, and washed with PBS twice. Preparation of Alg-HM culture system: A high-voltage DC power supply and a micro-sampling pump were prepared. The voltage was adjusted to 12 kV, the injection speed was 5 mL/h, and the inner diameter of the nozzle was 160 μm. The beaker was filled with a 0.1 M CaCl_2_ solution, and the prepared hMSC aggregates were added to the alginate solution for resuspension to form an hMSC aggregate suspension. Then, the hMSC aggregate suspension was added to a 10 mL nozzle tube, and a 160 μm stainless steel flat nozzle was used with a microinjection pump. After electrospraying, the liquid in the beaker was transferred to a 15 mL centrifuge tube, balanced, and centrifuged at 600 rpm for 2 min, and the supernatant was discarded. Preparation of Matrigel culture system: Matrigel (CORNING, Shanghai, China) and crushed ice were placed in a refrigerator at 4 °C to melt overnight, and pre-cooled serum-free culture was added to suspend hMSC aggregates; then, the hMSC aggregate suspension was diluted with the Matrigel at a ratio of 1:1, slowly added into a 96-well plate, placed in a 5% CO_2_ incubator at 37 °C for 30 min to solidify, and cultured with fresh DMEM/F12 medium, and the supernatant was discarded.

### 4.4. The Cell Counting Kit-8(CCK-8) Assay Was Used to Determine Cell Viability

Cell viability was determined using the CCK-8 assay on days 1, 3, 5, 7, and 14. In total, 100 μL of the CCK-8 reagent (Beyotime Biotechnology, Shanghai, China) was added into different culture wells and incubated for 2 h; then, 80 μL of the culture was taken from each well and transferred to a new 96-well plate. The absorbance density (OD) of each well was measured at 450 nm using a multifunctional ELISA reader (Thermo Fisher, Shanghai, China), and the cell proliferation rate was calculated using the OD value of each culture system on the first day as the initial value.

### 4.5. Calcein-AM/PI Cell Staining

The constructed hMSC aggregates were cultured in different culture systems in a 5% CO_2_ incubator at 37 °C until the 14th day. Calcein-AM/PI staining was performed on days 7 and 14; hMSC aggregates from different culture systems were collected and placed in 1.5 mL EP tubes; and the Tip-D group and Alg-HM were first dissolved in a 0.1 M EDTA solution for 5 min to remove the alginate coating. The Matrigel group was first melted at 4 °C and centrifuged at 600 rpm for 2 min to remove the gel. The cell aggregates were washed three times with PBS, incubated in the dark with 500 μL of the Calcein-AM/PI cell staining solution (Elabscience, Wuhan, China), incubated in a 5% CO_2_ incubator at 37 °C for 1 h, and then fixed in 4% paraformaldehyde for 30 min; the supernatant was discarded and washed twice with precooled PBS. A PBST solution was added and sealed at room temperature for 3 h, during which the centrifuge tube was inverted once every 30 min. It was then washed twice with PBSB and stained for 15 min with 50 μL of a DAPI solution containing an anti-fluorescence quencher (SouthernBiotech, Birmingham, AL, USA). Images of the cell aggregate staining were obtained using a laser confocal microscope (Nikon, Tokyo, Japan). The fluorescence intensity of each group of dead cells was quantified using ImageJ-1.54p software.

### 4.6. Ki-67 Cell Proliferation Detection

Proliferation was detected using Ki-67 staining on days 7 and 14. hMSC aggregates from different culture systems were collected and placed in 1.5 mL EP tubes; the Tip-D group and Alg-HM were first dissolved in a 0.1 M EDTA solution for 5 min to remove the alginate coating. The Matrigel group was first melted at 4 °C and centrifuged at 600 rpm for 2 min to remove the gel. The cells were washed twice with PBS, fixed on ice with 4% paraformaldehyde for 30 min, and washed thrice with PBSB. A normal goat serum solution (Biosharp, Anhui, China) was added and sealed at room temperature for 3 h, during which the centrifuge tube was inverted once every 30 min. After removing the immunostaining blocking solution, the Ki-67 rabbit monoclonal antibody (Beyotime Biotechnology, Shanghai, China) was added, the cells were incubated at 4 °C for 24 h, the Ki-67 rabbit monoclonal antibody was recovered, the cells were washed with PBSB three times, anti-rabbit Cy3 was added, the cells were incubated at room temperature in the dark for 3 h, and the tubes were inverted once every 30 min. The anti-rabbit Cy3 was recovered, the cells were washed twice with PBSB, and 50~100 μL of a DAPI solution containing an anti-fluorescence quencher was added for 15 min. A laser confocal microscope was used to obtain a stained image of the cell aggregates, and the fluorescence intensity of Ki-67 in each group was quantified using ImageJ-1.54p software.

### 4.7. RNA Sequencing Analysis

Total RNA was isolated as described above and was used for RNA-seq analysis. cDNA library construction and sequencing were performed at the Beijing Genomics Institute using the BGISEQ platform. High-quality reads were aligned to the human reference genome (GRCh38) using Bowtie2-2.5.4. The expression level of each gene was normalized to fragments per kilobase of exon model per million mapped reads (FPKM) using RNA-seq with Expectation Maximization (RSEM). A significant differential expression was set if a gene had a >2-fold expression difference compared to the control with an adjusted *p*-value of <0.05. The enrichment degrees of differentially expressed genes (DEGs) were analyzed using the Kyoto Encyclopedia of Genes and Genomes (KEGG) annotations.

### 4.8. Cell Cycle Assay

Cell cycle detection was performed using the cell cycle detection kit (Beyotime Biotechnology, Shanghai, China), following the manufacturer’s instructions. The specific steps are as follows: Briefly, cells were collected, washed with PBS, and centrifuged to remove the supernatant. Then, 300 μL of PBS was added, followed by 700 μL of anhydrous ethanol, drop by drop, to make it 70%, shaking while dropping. Cells were soaked in 70% ethanol overnight at 4 °C. The next day, the cells were washed with PBS and centrifuged to remove alcohol. Then, the cells were stained with propidium iodide staining solution (PI) and RNase A, and incubated in the dark at 37 °C for 30 min. The cells were analyzed using a flow cytometer (cytoFLEX LX, Shanghai, China) and Modfit LT 5.0 software. At least 1 × 10^4^ events were recorded per sample.

### 4.9. Statistical Methods

All experimental data were statistically analyzed using GraphPad Prism 10.1 software, and the results were expressed as SEM ± SD. Statistical differences were analyzed using two-way ANOVA, and each group of experiments was independently repeated three or more times (* *p* < 0.05, ** *p* < 0.01, *** *p* < 0.001).

## 5. Conclusions

In this study, a three-dimensional cell aggregate culture method based on alginate hydrogel was constructed using electrostatic spraying technology. The resulting Alg-HM culture system had a uniform particle size distribution, which can improve the nutrient transfer of hMSC aggregates in culture, significantly enhance cell proliferation activity, and reduce the degree of central necrosis. This culture system is suitable for in vitro culture, enabling detailed study of cell aggregates such as organoids, and contributes to efficient in vivo transplantation of stem cell aggregates. However, the effects of alginate hydrogel on mesenchymal stem cells from other sources need to be further verified. Additionally, the 14-day culture period used in this study is still too short for long-term culture, so the applicability of this system for long-term culture requires further validation. Despite the technical challenges in using alginate hydrogel as a delivery medium for stem cell aggregates, it is gradually becoming one of the most promising choices to simulate the three-dimensional natural matrix in the development of in vitro models.

## Data Availability

The original contributions presented in this study are included in the article. Further inquiries can be directed to the corresponding author(s).

## Supplementary Materials

The following Supporting Information can be downloaded at: https://www.mdpi.com/article/10.3390/ijms26136435/s1.
