# Supplementary material for: Construction of Microsphere Culture System for Human Mesenchymal Stem Cell Aggregates
Source: Int J Mol Sci. 2025 Jul 4;26(13):6435. doi: 10.3390/ijms26136435 (PMC12250013; doi:10.3390/ijms26136435)
Supplement: Supplementary file 1 [file ijms-26-06435-s001.zip › ijms-3687474-Supplementary.pdf]

Supplementary

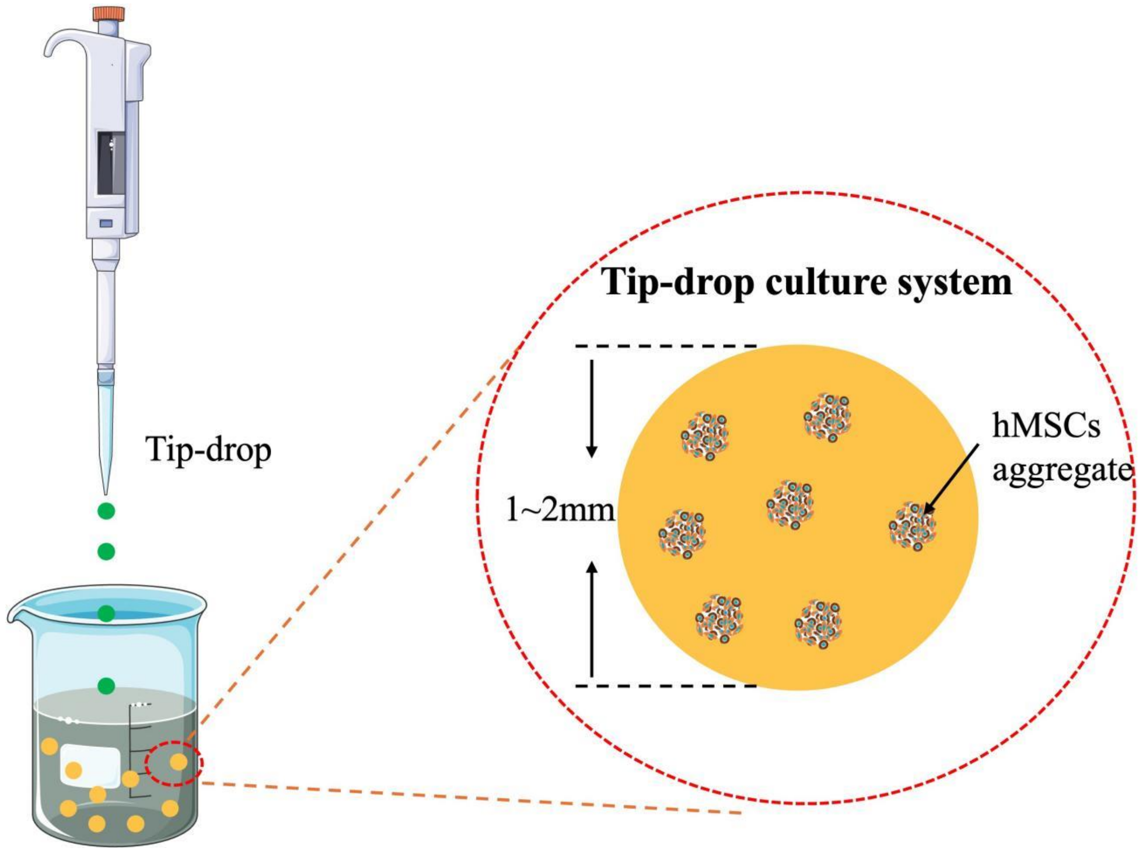

FigureS1. Schematic diagram of Tip-D culture system

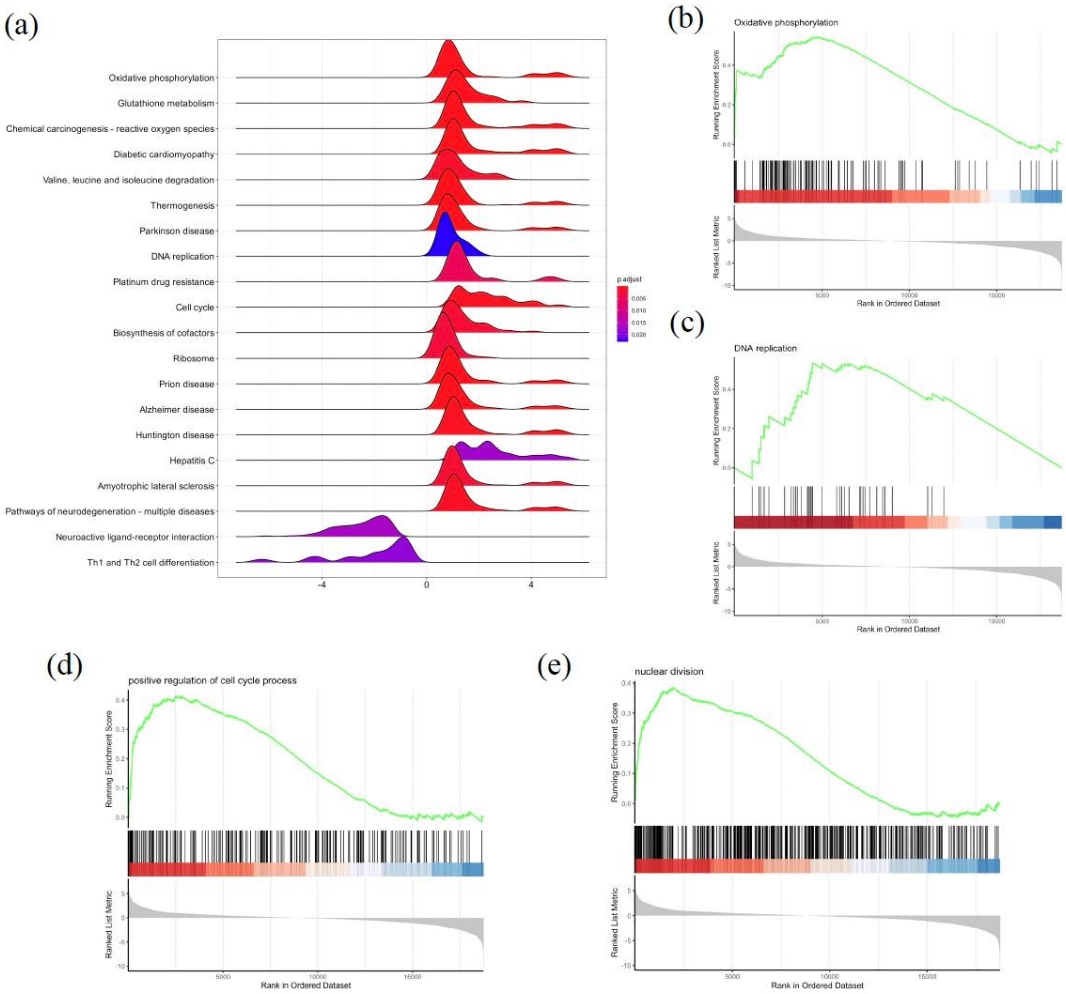

Figure S2. The gene set enrichment analysis (GSEA) of the DEGs of Alg-HM vs Tip-D at 7 days. (a) Ridge plot illustrating the distribution and expression of the gene set across different clusters (The horizontal axis is the enrichment score (ES) and the vertical axis is the enriched pathway; ES value greater than 0 indicates that the pathway is activated and the enriched core genes are up-regulated genes, ES value less than 0 indicates that the pathway is inhibited and the enriched core genes are down-regulated genes) (b) The gene set enrichment analysis (GSEA) of the gene related to oxidative phosphorylation (c) The gene set enrichment analysis (GSEA) of the gene related to DNA replication (d) The gene set enrichment analysis (GSEA) of the gene related to positive regulation of cell cycle process (e) The gene set enrichment analysis (GSEA) of the gene related to nuclear division.
